# Acknowledgment to Reviewers of *Bioengineering* in 2021

**DOI:** 10.3390/bioengineering9020050

**Published:** 2022-01-26

**Authors:** Bioengineering Editorial Office

**Affiliations:** MDPI AG, St. Alban-Anlage 66, 4052 Basel, Switzerland

Rigorous peer-reviews are the basis of high-quality academic publishing. Thanks to the great efforts of our reviewers, *Bioengineering* was able to maintain its standards for the high quality of its published papers. Thanks to the contribution of our reviewers, in 2021, the median time to first decision was 18 days and the median time to publication was 44 days. The editors would like to extend their gratitude and recognition to the following reviewers for their precious time and dedication, regardless of whether the papers they reviewed were finally published:
A. Sofia SilvaKristina Amalevičiūtė-VolungėAbdul AhadKristina HaaseAbhinav JainKristopher A. KilianAdam MasłońKrzysztof SztankeAdrian ArrietaKuangwen HsiehAdrian NeaguKumar Chandan SrivastavaAdriano TaddeoKun LiangAgata JanowskaKunihiko HiramatsuAgneta SimionescuKurt PfannkucheAgnieszka TomaszewskaKyriaki KyriakidouAgnieszka Ulatowska-JarżaKyung-Mee ParkAgustín ArandaLaith AlzubaidiAgustin L. Herrera-MayLaura MegidoAgustin ProbanzaLaura Suter-DickAhmed Al-SabiLeigh KotzéAjay K. WakhlooLenka LhotskaAkhlaq MuhammadLennart HardellAkinrinade George AyankojoLi LinAlaa A A AljabaliLi YangAlejandro Roldán-AlzateLiang MaAleksandr BulaevLiangsong HuangAleksandra Piechota-PolanczykLiangwei DengAleksandra V. BukhovetsLifeng KangAlessandro CataniaLikang ChinAlessandro F. MartinsLina BaranauskieneAlessandro GiudiciLiwei LiuAlexander G. MathioudakisLluís BlanchAlexander OtahalLong LiuAlexandru MesterLorena Del Pozo-AceboAlfredo Cabrera OreficeLouise WrightAli FarziLuca AmbrosioAli ZarrabiLuca FalzoneAlicia El HajLuca TestarelliAlok SutradharLucian Toma CiocanAlvaro ValenciaLudovica CacopardoAmiq GazdharLuis CoelhoAmir M. Fathollahi-FardLuis G. C. PachecoAmir MosaviLu-Ting KuoAmit JaiswalLydia Gimenez LlortAna DávilaMa Del Rocio López-CuellarAna KramarMafalda MassaraAnand ThirupathiMaha NasrAnastasia A. AnashkinaMahmoud WagihAnca M PascaMahsa DabaghAndrea De PieriMałgorzata Nattich-RakAndrea Dell’AmoreMałgorzata Szultka-MłyńskaAndrea GarattiMangesh HadeAndreas KolkManisha PandeyAndreas KremlingManoj GargAndreia SalvadorManoj KumarAndrey KaplanMans BroekgaardenAndrzej PolańczykManuel Albornoz-CabelloAngela LongoMarc ChansonAnna DrabczykMarc Van HulleAnna Garus-PakowskaMarco CicciùAnna MichnikMarco FarronatoAnna RichelliMarco TatulloAnna SkorskaMarcus L. F. JanssenAnna SurdackaMargherita CortiniAnnika MutanenMaria Leilani TorresAnthony L. PanosMaría Orosia Lucha-LópezAnton FicaiMaría S. ÁlvarezAntonia KaltsatouMaria Valentina DinuAntonio ApicellaMaría Virumbrales-MuñozAntonio E. UvaMarica MarkovicAntonio Giovanni SolimandoMario Alberto García RamírezAntonio GloriaMario RothbauerAntonios GiannopoulosMarjan BoermaAnvay UkidveMarkus A. HartmannAnya GrosbergMarkus VeltenApostolos I. TsolakisMarta KlakAranzazu VillasanteMartin EnglundAre Hugo PrippMartin R. PfallerArkadiusz JózefczakMartina ChiuArnaldo Rodrigues Santos JúniorMartina MugnanoÁrpád Ferenc KovácsMartine PithiouxArturo Ibáñez-FonsecaMarwa El SouryAshwini Rahul AkkineniMary C. Farach-CarsonAtefeh SoloukMaryam DavaritouchaeeAthanasios GoulasMasamitsu IchihashiAvinash WaghrayMasoud MozafariBabagolzadeh MahlaMassimiliano PanellaBarbara CerroniMassimo CapocciaBarbara RuffinoMassimo CorsaliniBart PijlsMateusz KowalikBartłomiej ZieniukMathias TrempBenyamin KhoshnevisanMatia MainardisBeom Jin KimMatthew WadeBernardo Martins RochaMauro MalvèBernhard WernlyMauro MarzoratiBertrand LiagreM.Carmen Martinez-GarciaBhavani S. KowtharapuMd. Abdul AwalBiagio SantellaMd Abdus SubhanBikash R. PattnaikMegan N. BallingerBipin GaihreMehdi MehraliBiraja DashMeral YüceBirgit AndréeMercedes GinerBoris SchmittMichael SchweizerBrent SieskyMichael TrembleyBrian E. GrottkauMichalis KatsimpoulasBruno ChrcanovicMichele CassettaByungheon LeeMichele Christian KlymiukByungsuk KwonMichele LanzaC. Henrique AlvesMieszko WieckiewiczC. Noel Bairey MerzMiguel A OrtegaCalin VaidaMiguel Angelo SchettinoCambón AdrianaMiguel Vicente-ManzanaresCarl AlvingMilan KoszelCarlo GrecoMilena Álvarez-ViñasCarlotta PontremoliMiloš MilovanovićCary KuliashaMin GuanCaterina CrediMinaxi SharmaCecile LegallaisMinliang LiuCécile MartinatMinseok KwakCeline HenoumontMiralda I. BlinovaChaenyung ChaMiroslav MrlíkChander PrakashMohamad SawanChao WuMohamed A. HassanChelliah V. NavinMohamed M. Abdel-DaimChen XuMohammad NorouzifardCherie KrugerMonica De PalmaChiara Giulia FontanellaMonica FlorescuChih-Hao WangMonica ScrieciuChihyang WangMonika HertenChin-Lin GuoMonika RatajczakChiuan-Chian ChiouMonika Sakowicz- BurkiewiczChristian G. PfeiferMorteza Hasanzadeh KafshgariChristina ToftMotta SarahChristophe HélaryMudasir Bashir GugjooChristopher M. MadlMuhammad NawazChun Hung ChuMuhammad QasimChung-Kan PengMuhammad Wajid UllahCian O’LearyMunna KhanCinzia MasperoMurali MaruthamuthuClaire-Marie RangonMuthu ArumugamClara García-AstrainMyeongkee ParkClaudia Sandoval-YáñezNancy GavertCodruta VarodiNaoaki SakataCortino SukotjoNapoleão Martins Argôlo NetoCristina NastasaNark-Kyoung RhoCrystal S ShinNarrendar RaviChandranDamian HudziakNathan CrookDamien DupinNavaneeth Krishna Rajeeva PandianDan SimionescuNeelesh Kumar MehraDana BadauNeil BressloffDaniela BuchaimNicholas R. HumDaniela Valdez-JassoNicoleta AntonDanny Van NoortNicoleta RaduDariusz T. MlynarczykNiel Van WykDavi Marco Lyra LeiteNiels Holten-AndersenDavid L. MayorNien-Ti TsouDavid Ortiz De ZárateNikhlesh SinghDeclan O’LoughlinNikita MinaevDe-Li ShiNizar B. AlsharifDennis DouroumisNobue ItasakiDhamodharan VenugopalNoemi De Luna SalvàDiana MassaiNongyue HeDiana PortanNorbert ZolekDiego Alexander Tibaduiza BurgosNorma-Aurea Rangel-VázquezDiego VelascoNorman R. WilliamsDimitrios ZeugolisOana RasogaDinesh Kumar PatelOlalla Iglesias-GarcíaDinesh RokayaOleg LookinDirk KucklingOlga KopachDjordje VeljovicOliver GembruchD.M. CorreiaOlivier DeschaumeDmitrii KaplunOmid Haji MaghsoudiDmitry ValeevOmid KhalajDomenica Donatella Li PumaOmri EmodiDomenico MaisanoOriana TrubianiDominik ObristÓscar Barquero-PérezDong LiOskar SachenkovDong LiuPablo G. Del RíoDragoljub GajicPak ChanEddy KruegerPanagiota KatikouEkaterina BaryshnikovaPanagiotis MallisElaine TangPankaj DhatrakElena StoccoPaolo CrippaElham AhmadianPaolo NevianiElias AugustPaolo RebullaElisabetta M ZanettiPaolo ViscontiElizabeth S. DylkeParag KatiraElmina Mammadova-BachPascal ColosettiEmi YudaPasquale MarrazzoEmilia ManolePatricia ArancibiaEoghan CunnanePau RedonÉric GrivelPaul LindahlEric LevesquePaula Fraga-GarcíaErminda TsoukoPavana RottiEsmaeel SharifiPawel NakielskiEstíbaliz AlegrePeshala GamageEustace Y. FernandoPetra SurlinEwa KorzeniewskaPetronilla BattistaEwelina Świa̧tek-NajwerPhilip GardinerFabiana NicitaPia Lopez-JornetFabrizio Di CaprioPierpaolo Di MiccoFang ChenPierre BourgeoisFany ReffuveillePierre-Yves Collart-DutilleulFardin KhaliliPiyush BaindaraFarokh DemehriPiyush KoriaFatima A. SalehPradeep KumarFei XuPraveen BommareddyFeng QiPrzemysław DorożyńskiFiona LouisQasim AlhadidiFiras KobeissyQi GuFlorin CiolacuQiao LiFnu Ravinder KumarQuang NguyenFrancesca Boscolo SesilloR. Gary SawersFrancesca CosmiRajendran JC BoseFrancesca GaffuriRajiv Ranjan SrivastavaFrancesco BainoRamaswamy KannappanFrancesco MaininiRamesh S YadavaFrancisco Sahli CostabalRamya Lakshmi RajendranFrancisco Vega ReyesRan MeiFranck JourdanRandolph D. GlickmanFrançois KuonenRashid AminFrank KoRaul Torres-RuizFrank PetriglianoRawen SmiraniFull NameRenata TeparićGabriel FurtosReza AvazmohammadiGaetano SantulliRicardo VardascaGagan FloraRiccardo BeltramiGaia ManninoRobert A.F.M. ChamuleauGaia PellegriniRobert KellyGautam MahajanRobert P. MechamGenki TerashiRobertas DamaševičiusGennaro MartucciRoberto GramignoliGeorge A. PapakostasRoberto Portillo-LaraGeorge A TruskeyRochelle AwGeorge Vladimirovich SharonovRocio Litran RamosGeorge Z. PapageorgiouRodolfo RedaGeorges BaffetRodrigo Lira De OliveiraGerald F AudetteRoger J. GuilloryGiada GiorgiRomána ZelkóGilbert LimRosa CibriánGiorgia AilunoRuben San-SegundoGiorgio ColomboRupesh K. MishraGiovanni TarantinoRyoko Takao-KawabataGiovanni VozziS. Jun SonGiuseppe Lo GiudiceS. M. J. RazaviGiuseppe SantarpinoS. S. SimakovGlen MartinSabine SzuneritsGolnaz KaroubiSabrina MorelliGöran LindSadanand PandeyGowthamarajan KuppusamySaeid KargozarGraziano MontaruliSalvatore A. PullanoGrzegorz RedlarskiSamira MalekmohammadiGrzegorz TrybekSandeep KeshavanGu LiSandipan RoyGuangli LiSandra BravoGuanqiu QiSantiago Perez-LloretGulsim KulsharovaSara ChiappalupiGuoan LiSayan BasuGuya Diletta MarconiSayan GangulyGuzel ZiyatdinovaSean ChambersHaigang WuSean Robert AsselinHaiwei XuSebastian L. RiedelHajime UtsunomiyaSébastien PigeotHamed Salimi-KenariaSejoong KimHan Eol LeeSelvakumar SubbianHang SuSenol PiskinHanieh KhaliliSentaro KusuharaHarikrishnan ParameswaranSeong-Gon KimHarikumar RajaguruSerge AbrateHaris NalakathSergey A. SinenkoHarish ChandramoorthySergey SedykhHarvey R. FernandezSergio CasellaHassan RashidiSergio SambataroHaveman Jan WillemSérgio VelosoHee Ho ParkSerguei SekatskiHelena RitchieSha ShaHermis IatrouSheng-Dong XuHesam KamyabShengkun XieHetong YangShin Jun ParkHiroki YokotaShingo IzawaHo Seong SeoShintaro SukegawaHojong ChoiShivshankar ThanigaimaniHongbing DengShizhou WuHongpeng ZhangSiddhartha MahantyHongqiang WangSigrid A. LanghansHongwei LiSimon W. RabkinHongwei OuyangSiti BaidurahHorea FeierSmaoui SlimHsu MaSoja SomanHuanyu (Larry) ChengSolomon DiamondHugh DevlinSophie MartinHugo Fernando Posada-QuinteroSougata GhoshHugo López FernándezSpencer E. BlivenHuu Hao NgoSpyridon NtougiasHwan D. KimSpyros ManolopoulosHyemin KimSrecko StopicHyo-Eon JinStanescu Ana Maria AlexandraHyoSung KwakStefan WandererHyun Jong LeeStefano LeporattiIbrahim JavedStela-Maria PRUNEANUI-Chi LeeSten AnslanIgor DjurovićStéphane ChabaudIgor VujovićSteve ElderIhtesham RehmanStuart FraserIlaria BortoneStuart TobetIlya KlabukovSuzanne MithieuxIman YazdiSwee Leong SingIndrajit RoyTae-Eun ParkInês FranciscoTaisiya SigaevaInga MarijanovicTakashi HoshibaInna MelnykTakayuki KawatoIoannis TheologidisTakayuki SekitoIonut-Cristian RaduTakuya TaniguchiIriczalli Cruz-MayaTamako HataIris RibitschTamara ReinickeIsha MutrejaTanja ZidaričIsmail FidanTanvir FaisalIsrael González De TorreTarik ArafatIssei OtsukaTariq MalikJacek HoffmanTatyana V. AbramovaJacek MatysTaylor J. ReifJaison JeevanandamTed S. AcottJakub GrzesiakTeresa RussoJakub TaradajTetsuo SasanoJames E. MooreTheodoros RampiasJames JohnsonThomas A. TraboldJames OttonThomas DistlerJames W. FosterTiago DiasJamir Pitton RissardoTimothy HughesJan EggerTina MaverJan PorzuczekTissiana BortolottoJanith WeerasingheTomasz RymarczykJanusz KarasińskiTomasz ŻokJaroslav HronTommaso IngrassiaJaroslaw DrelichTorsten SchenkelJaroslaw MellerTzu-Ching ChangJasmina Casals TerreTzu-Ching ShihJavad AlizargarUlrike WittigJavad TavakoliUpul GunawardanaJavier Ramón-AzcónVahid SerpooshanJay D. KeaslingVaibhav JainJe Hyun SeoVan Giau VoJeevithan ElangoVanessa Andrés-GuerreroJeffrey GimbleVarun JeotiJennifer PuetzerVenkatraman GopinathJeong Chan JooVera FaustinoJeonghyun KimVeronika AleksandrovychJeroen RoelofsVeronika PavliňákováJérôme DuisitVickery Trinkaus-RandallJérôme LeprinceVíctor Manuel Pérez PuyanaJerzy BiałeckiVictor UjorJessica YoungVijayavenkataraman SanjairajJesús MingoranceVikas Kumar SangalJiabing FanVincent SallesJiajia XuVincenzo Di StefanoJiajia XueVincenzo GrassiaJianhua ZhangVinod SureshJiao Jiao LiVitor GeraldesJiaquan YuVladimir N. Sil’nikovJinao ZhangVuk UskokovićJingshu GuoW. D. GrimmJin-Jia HuWafaa ShalashJiong LiWalter P. PflieglerJiranuwat SapudomWarren BluntJiro NagatomiWei SunJitendra KanaujiyaWeifeng LinJitendra Kumar SainiWenguo CuiJoakim HakanssonWen-Song TanJoan MonteroWerner BaumgartnerJoana Vieira De CastroWiktor NiewiadomskiJoanna MichalowskaWilliam J. FederspielJoão H. P. M. SantosWilliam ZimmermanJoão-Paulo-Mendes TribstWolf Hans EilenbergJochen SalberXi LouJohn GreenmanXiaojing LiJonathan GrasmanXin-Luan WangJordan CicilianoXuegang YuanJörg KretzschmarXuenan GuJorge N.R. MartinsXufeng XueJosé Javier Reyes-LagosYan WangJose Maria Pereira De GodoyYanfei QiJose RodellarYannis KaramanosJuan C OliverosYasumoto NakazawaJuan M. GandariasYeong-Jin ChoiJudith M. BragancaYiqian HeJuei-Tang ChengYoo Min ParkJui-Che LinYoshifumi SaijoJun KobayashiYoshihiro ToyaJung-Jae LeeYoshimi SeidaJunil KimYousif MohammedJustyna A. NiestrawskaYuancong XuJuvenal Rodríguez-ReséndizYujia XuK. KisandYuksel TemizKamal Kant SahuYuling YanKamchai NuithitikulYuly A. Ramirez-TapiasKapil PatelYundi FengKatarina MihajlovskiYunqing (Kevin) KangKatarzyna Bialik-WąsYves BayonKatia BarbaroZacharoula IatridiKazutoshi IijimaZdenka Zakova KroupovaKazuyuki ShimizuZehong CaoKelvin AfrashtehfarZehuan LiaoKetan M. RanchŽelimir KurtanjekKevin DzoboZenon LukaszewskiKhaled GiasinZhaosheng HouKinga BociongZhenqi JiangKiran ChereddyZhiming RaoKlementieva Natalia V.Zhiquan ShuKoji FujitaZhong LiKoji OtaniZhongkui HongKonstantin Johannes ScholzZihao OuKoroush KabirZimi SawachaKrishna Mohan Padmanabha DasZuhairiah Zainal AbidinKrist V. Gernaey



